# Impact of microRNA-29b on nature killer cell in T-cell acute lymphoblastic leukemia

**DOI:** 10.3892/ol.2020.12273

**Published:** 2020-11-05

**Authors:** Fengyan Jin, Zhonghua Du, Yang Tang, Lixia Wang, Yanping Yang

Oncol Lett 18: 2394-2403, 2019; DOI: 10.3892/ol.2019.10559

Following the publication of this article, the authors have realized that a couple of errors went unnoticed in the text. These are explained as follows (changes to the printed text are highlighted in bold):

In the “Statistical analysis” section, on p. 2396, the 7^th^ line, it was incorrectly stated that the RT-qPCR data were analyzed using SPSS 12.0 (SPSS, Inc.); in fact, Prism 5 (GraphPad Software, Inc.) was also used for the statistical treatment of these data. Therefore, the sentence here in its entirety should have read as: “The flow cytometry results were analyzed using FlowJo 10.0.7 software (FlowJo LLC), and the RT-qPCR data was analyzed using **Prism 5 (GraphPad Software, Inc.)**” This error does not affect the results in any way.

Secondly, the authors have realized that, on p. 2395 in the “Animals” subsection of the Materials and methods, they overlooked providing details of the source of the C57BL/6 miR29ab1^−/−^ mice. The C57BL/6 miR29ab1^−/−^ mice were provided as a gift from Shanghai Model Organisms Center, Inc. The authors regret that these errors were allowed to remain in the text, and thank the Editor of *Oncology Reports* for allowing them to publish this Corrigendum.

